# Supplementary material for: The Post-Curing of Waterborne Polyurethane–Acrylate Composite Latex with the Dynamic Disulfide-Bearing Crosslinking Agent
Source: Molecules. 2023 Dec 15;28(24):8122. doi: 10.3390/molecules28248122 (PMC10745677; doi:10.3390/molecules28248122)
Supplement: Supplementary file 1 [file molecules-28-08122-s001.zip › molecules-2722876-supplementary.pdf]

Supporting Information

The Post−Curing of Waterborne Polyurethane−Acrylate Composite Latex with the Dynamic Disulfide−Bearing Crosslinking Agent

Haotian Zhang,[1,2] Rihui Liang,[1,2] Qianshu Wang,[1,2] Wenbo Luan,[1,2] Jun Ye [1,2,*]
Teng Qiu [1,2,*] & Xinlin Tuo [3]

1 Key Laboratory of Carbon Fiber and Functional Polymers, Ministry of Education, Beijing University of Chemical Technology, Beijing 100029, China

2 Beijing Engineering Research Center of Synthesis and Application of Waterborne Polymer, Beijing University of Chemical Technology, Beijing 100029, China

3 Key Laboratory of Advanced Materials (MOE), Department of Chemical Engineering, Tsinghua University, Beijing 100084, China

[*]Corresponding authors:
Teng Qiu (qiuteng@mail.buct.edu.cn); Jun Ye (yejun@mail.buct.edu.cn)

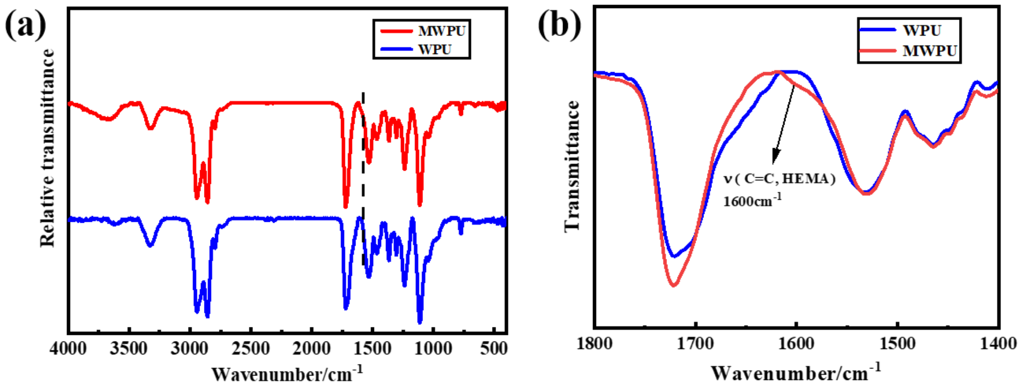

Figure S1. (**a**) The FTIR spectra of MWPU and WPU. (**b**) The zoom-in spectra of MWPU and WPU.

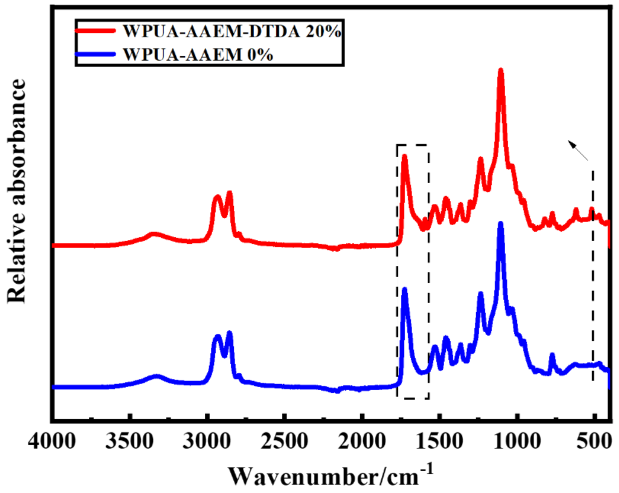

Figure S2. ATR-FTIR spectra of WPUA-AAEM 0% and 20% films.

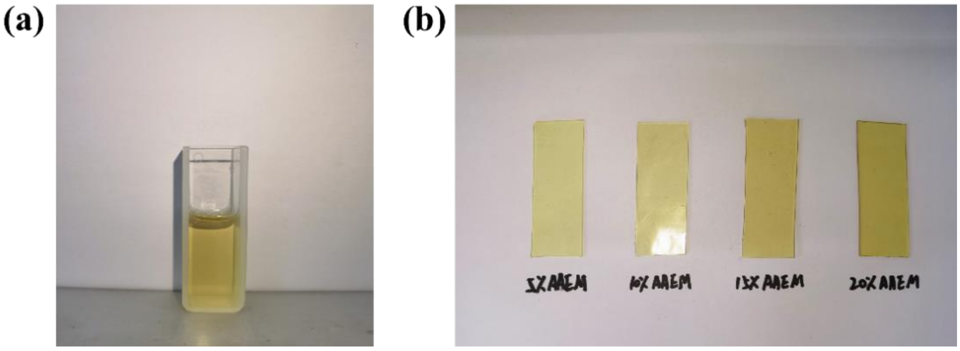

Figure S3. (**a**) The photographs of the ethanol solution of DTDA. (**b**) The photographs of WPUA-AAEM-DTDA *x*% films.

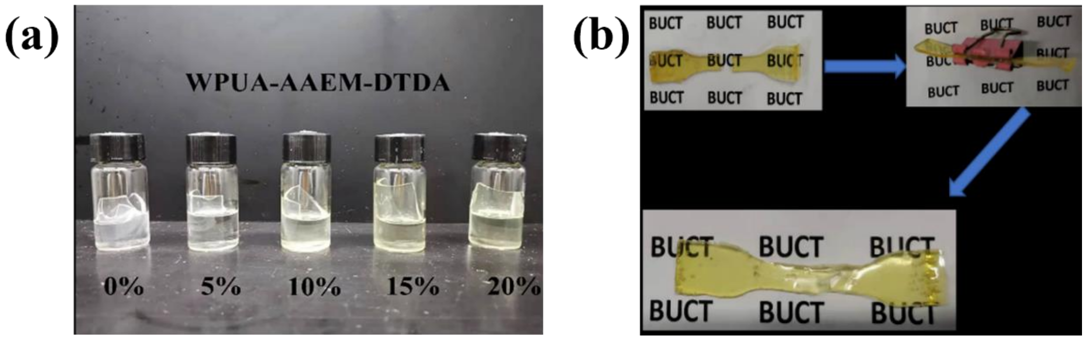

Figure S4. (**a**) The states of WPUA-AAEM-DTDA *x*% films after 24h of immersion in THF. (**b**) Self-healing process of WPUA-AAEM-DTDA 20% film.

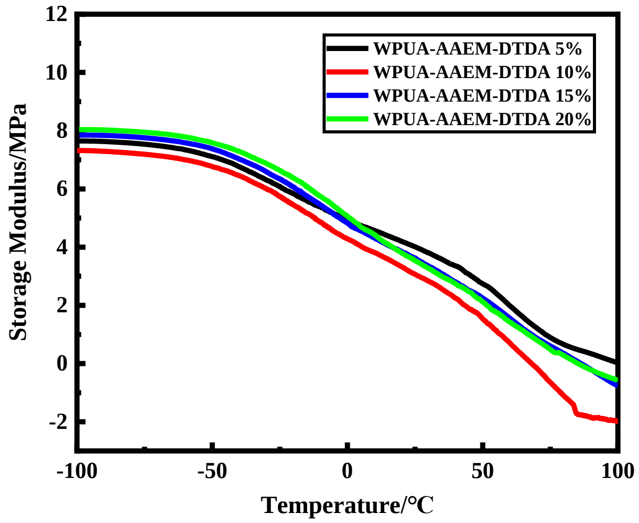

Figure S5. Storage modulus-temperature curves of the WPUA-AAEM-DTDA *x* % films.

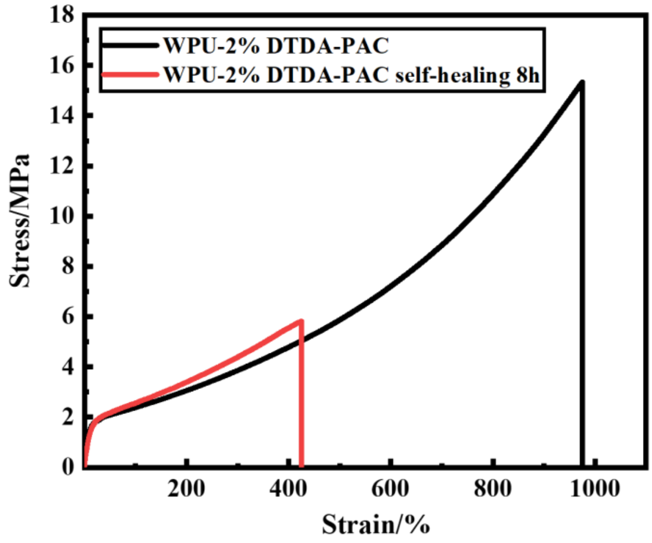

Figure S6. Stress-strain curves of the films self-healed at 70°C for 8 hours in comparison with the original films of WPU-2% DTDA-PAC.

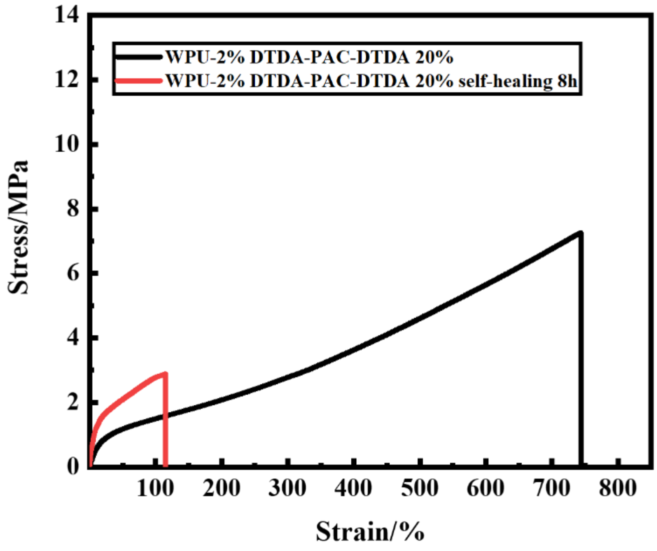

Figure S7. Stress-strain curves of the films self-healed at 70°C for 8 hours in comparison with the original films of WPU-2% DTDA-PAC-DTDA 20%.
